# Supplementary material for: Kernel density estimation of allele frequency including undetected alleles
Source: PeerJ. 2024 Apr 22;12:e17248. doi: 10.7717/peerj.17248 (PMC11044881; doi:10.7717/peerj.17248)
Supplement: Supplemental Information 2 [file peerj-12-17248-s002.docx]

Appendix

**Frequency and distance between a detected allele and undetected alleles**

Frequency of detected alleles can be directly obtained from the result of KDE. When the component of the coordinate of an undetected allele is not “Inf”, then the frequency of the undetected allele is also directly obtained as well as the distance between the detected allele and undetected allele. The problem is when the corresponding component is recorded as “Inf”. In this case, both of the frequency and distance cannot be directly obtained and the following shows how to obtain them.

Consider a group of alleles which are with the length $l$ and $x$ distance away from an allele A and name the group as $A_{x}$. The number of alleles belonging to $A_{x}$ is referred as $\#A_{x}$, and $\#A_{x}={}_{l}{P_{x}3^{x}}.$ Next, consider another allele B with the length $l$ that is $d$ distance away from the allele A, and name a group of alleles $B_{d+y}(A_{x})$, which belong to $A_{x}$ and are $d+y$ distance away from the allele B (Figure S1). The number of alleles belonging to $B_{d+y}(A_{x})$ is referred as $\#B_{d+y}(A_{x})$. Here, the allele B corresponds to a detected alleles and $A_{x}$ corresponds to undetected alleles.

To start from the simplest situation, consider $A_{1}$. The allele A has $l$ bases, and $d$ of them are different bases from those of the allele B. Inevitably, $l-d$ of them are the same as the allele B. When a base in the allele A which is the same as the allele B mutates, the mutated allele is $d+1$ distance away from the allele B, and the total possible number of the mutated alleles is $3(l-d)$ because there are three different bases except the one before mutation. When a base in the allele A which is different from that of the allele B mutates, there are two patterns: When the mutated allele is the same as the allele B, the mutated allele is $d-1$ distance away from the allele B, and its total possible number is $d$. When the mutated allele is still different from that of the allele B, the mutated allele is $d$ distance away from the allele B, and its total possible number is $2d$. The above discussion is concisely expressed as the following equations:

$$\#B_{d-1}(A_{1})=d$$

$$\#B_{d}(A_{1})=2d$$

$$\#B_{d+1}(A_{1})=3(l-d)$$

Because distance between alleles changes one by one, $\#B_{d-1}(A_{1})$, $\#B_{d}(A_{1})$ and $\#B_{d+1}(A_{1})$ exhaustively correspond to all the $\#B_{d+y}(A_{1})$, which equals to $\#A_{1}$.

Next, $\#B_{d+y}(A_{2})$ can be calculated from the result of $\#B_{d+y}(A_{1})$. In $B_{d+1}(A_{1})$, $l-d-1$ bases are the same as the allele B and $d+1$ bases are different from those of the allele B. When the former mutates, the mutated alleles are $d+2$ distance away from the allele B. When the later mutates, again there are two patterns: When the base after mutation is the same as the allele B, the mutated alleles are $d$ distance away from the allele B. When the base after mutation is different from that of the allele B, the mutated alleles remains $d+1$ distance away from the allele B. This is expressed as the following equations:

$$\#B_{d}(A_{2})=\#B_{d+1}(A_{1})\cdot d$$

$$\#B_{d+1}(A_{2})=\#B_{d+1}(A_{1})\cdot2d$$

$$\#B_{d+2}(A_{2})=\#B_{d+1}\left( A_{1} \right)\cdot3\left( l-d-1 \right)$$

Likewise, total of the mutation in $B_{d+1}(A_{1})$, $B_{d}(A_{1})$ and $B_{d-1}(A_{1})$ will be the following equations:

$$\#B_{d-2}(A_{2})=d(d-1)$$

$$\#B_{d-1}(A_{2})=2\cdot2d(d-1)$$

$$\#B_{d}(A_{2})=2\cdot3d\left( l-d \right)+2\cdot2d(d-1)$$

$$\#B_{d+1}(A_{2})=2\cdot3\cdot2\cdot d\left( l-d \right)$$

$$\#B_{d+2}(A_{2})=3^{2}(l-d)(l-d-1)$$

As shown above, the coefficient changes depending on the number of bases which are the same/different as/from that of the allele B. Now, mutation of a base in the allele A which is the same as the allele B is referred as $\alpha$, mutation of a base in the allele A which is different from that of the allele B into a base different from that of the allele B is referred as $\beta_{1}$, and mutation of a base in the allele A which is different from that of the allele B into a base same as the allele B is referred as $\beta_{2}$. Then, $\#B_{d+y}(A_{2})$ shown above can be expressed by the following way:

$$\#B_{d-2}(A_{2}): \beta_{2}\beta_{2}$$

$$\#B_{d-1}(A_{2}): \beta_{1}\beta_{2}+\beta_{2}\beta_{1}$$

$$\#B_{d}(A_{2}): {\alpha\beta}_{2}+\beta_{1}\beta_{1}+\beta_{2}\alpha$$

$$\#B_{d+1}(A_{2}): {\alpha\beta}_{1}+\beta_{1}\alpha$$

$$\#B_{d+2}(A_{2}): \alpha\alpha$$

The multiplication among $\alpha$, $\beta_{1}$ and $\beta_{2}$ is not commutative, and they correspond to the following coefficients:

$$\alpha: 3\left( l-d-\#\alpha^{'} \right)$$

$$\beta_{1}: 2(d-\#\beta')$$

$$\beta_{2}: (d-\#\beta')$$

Here, $\#\alpha'$ is the number of $\alpha$ before the corresponding multiplication, and $\#\beta'$ is the number of the sum of $\beta_{1}$ and $\beta_{2}$ before the corresponding multiplication.

Now, total numbers of $\alpha$, $\beta_{1}$ and $\beta_{2}$ are referred as $\#\alpha$, $\#\beta_{1}$ and $\#\beta_{2}$. Then, the following equations are satisfied:

$$x=\#\alpha+\#\beta_{1}+\#\beta_{2}$$

$$y=\#\alpha-\#\beta_{2}$$

Using the above expressions, sum of all the multiplications of $\alpha$, $\beta_{1}$ and $\beta_{2}$ provides$\#B_{d+y}(A_{x})$. For example, the multiplication $\alpha\beta_{1}\beta_{2}$ contributes to $\#B_{d}(A_{3})$ and the contribute is $3\left( l-d \right)\cdot2d\cdot(d-1)$.

Since the coefficients $\alpha$, $\beta_{1}$ and $\beta_{2}$ are not commutative, the results of their multiplications are not single; the permutation of $\alpha$, $\beta_{1}$ and $\beta_{2}$ is

$$\frac{x!}{\#\alpha!\#\beta_{1}!\#\beta_{2}!}.$$

Therefore, $\#B_{d+y}(A_{x})$ will be expressed by the following equation:

$$\#B_{d+y}(A_{x})=\left\{ \begin{aligned} \sum_{i=0}^{X} \{\frac{x!}{\#\alpha!\#\beta_{1}!\#\beta_{2}!}\cdot\alpha^{y}\beta_{1}^{x-2i-y}\left( \alpha\beta_{2} \right)^{i}\} \left( \mathrm{when} y\geq0 \right) \\ \sum_{i=0}^{X} \{\frac{x!}{\#\alpha!\#\beta_{1}!\#\beta_{2}!}\cdot\beta_{2}^{-y}\beta_{1}^{x-2i+y}\left( \alpha\beta_{2} \right)^{i}\} \left( \mathrm{when} y\leq0 \right) \end{aligned} \right.$$

$$=\left\{ \begin{aligned} \sum_{i=0}^{X} \{\frac{x!}{\left( y+i \right)!\left( x-2i-y \right)!i!}\cdot3^{y+i}\prod_{j=0}^{y+i-1} \left( l-d-j \right)\cdot2^{x-2i-y}\prod_{j=0}^{x-i-y-1} \left( d-j \right)\} \left( \mathrm{when} y\geq0 \right) \\ \sum_{i=0}^{X} \{\frac{x!}{\left( -y+i \right)!\left( x-2i+y \right)!i!}\cdot3^{i}\prod_{j=0}^{i-1} \left( l-d-j \right)\cdot2^{x-2i+y}\prod_{j=0}^{x-i-1} \left( d-j \right)\} \left( \mathrm{when} y\leq0 \right) \end{aligned} \right.$$

Here, $X$ is $x/2$ when $x$ is even and is $(x-1)/2$ when odd.

This $\#B_{d+y}(A_{x})$ provides the number and distance from the allele B of undetected alleles around the allele A. Therefore, even when a coordinate of undetected alleles $A_{x}$ is “Inf” distance from the detected allele B, the frequency of the coordinate of undetected alleles can be distributed to each group of alleles having actual finite distance to the allele B proportional to the number of undetected alleles. Scanning $x$ from 1 to the mutation number and scanning all pairs of the detected allele A and B provide all the terms between a detected allele and undetected alleles to calculate approximate nucleotide diversity.
